# Form and Function: The Factors That Influence the Efficacy of Nanomaterials for Gene Transfer to Plants

**DOI:** 10.3390/molecules30030446

**Published:** 2025-01-21

**Authors:** Zhila Osmani, Marianna Kulka

**Affiliations:** 1 Department of Medical Microbiology and Immunology, University of Alberta, Edmonton, AB T6G 2E1, Canada; marianna.kulka@nrc-cnrc.gc.ca; 2 Quantum and Nanotechnologies Research Center, National Research Council Canada, Edmonton, AB T6G 2M9, Canada

**Keywords:** nanoparticles, gene delivery, plant biotechnology, plant transformation, crop improvement, biocompatibility

## Abstract

Nanoparticle (NP)-mediated gene delivery offers a promising alternative to traditional methods in plant biotechnology, facilitating genetic transformations with enhanced precision and efficiency. This review discusses key factors influencing NP efficacy, including plant cell wall composition, DNA/NP ratios, exposure time, cargo loading, and post-transformation assessments. We explore the challenges of NP cytotoxicity, transformation efficiency, and regeneration while addressing environmental impacts and regulatory considerations. We emphasize the potential for stimulus-responsive NPs and scalable delivery methods to optimize gene editing in agriculture.

## 1. Introduction

The genetic modification of plants has revolutionized agriculture, enabling the development of crops with enhanced traits, such as disease resistance, drought tolerance, and improved nutritional content [1]. Traditional methods of plant transformation, such as Agrobacterium-mediated gene transfer and biolistic particle delivery, have been crucial in this evolution. However, these techniques are often limited by challenges, such as targeting specific tissues or cell types and the need for time-consuming and labor-intensive tissue culture [2,3,4]. As a result, there is a pressing need for innovative approaches to overcome these barriers and enhance the precision of genetic engineering.

Nanoparticle (NP)-mediated gene delivery is a precise and versatile technique, offering unique advantages over conventional methods. NPs have a high surface area, tunable size, and the ability to encapsulate diverse biomolecules, making them ideal candidates for gene delivery systems [5,6,7,8]. In order for NPs to be effective gene delivery systems, they must interact with plant cells such that they enter the cell and deliver their cargo. The interaction between NPs and plant cells is influenced by several factors, including the composition and structure of the plant cell wall, which can vary significantly across different species and tissues [9,10,11]. For example, some plant species have more porous cell walls that facilitate NP penetration, while others exhibit rigid and resistant structures that hinder the delivery of genetic material [12]. Understanding these variations is crucial for optimizing NP design and enhancing gene delivery efficiency.

Moreover, the efficacy of NP-mediated delivery is contingent upon several other parameters, including the DNA/NP ratio, exposure time, cargo loading mechanisms, and the NPs’ biocompatibility [8,13,14,15]. All of these parameters must be coordinated to ensure that sufficient genetic material is delivered without disrupting the chemical stability of the NP carriers [15,16]. The controlled release of biomolecules from NPs in response to specific stimuli is an exciting new area of investigation, potentially allowing for the precise control of gene expression in response to environmental conditions [12,15,17].

While NP-mediated gene delivery has a lot of potential applications, there are several challenges, particularly issues with cytotoxicity, transformation efficiency, and the regeneration of transformed plants [18,19]. Additionally, the environmental impact and regulatory considerations surrounding the use of nanomaterials in agriculture must be thoroughly addressed to ensure their safe application [20,21].

In this review, we will explore the current advancements in NP-mediated gene delivery, factors influencing its effectiveness, and challenges that need to be overcome for successful implementation in agricultural practices. By examining recent research and proposing future directions, we aim to provide a comprehensive overview of this exciting field and its potential to shape the future of plant biotechnology.

## 2. NPs as Plant Gene Delivery Vehicles

**Figure 1:**
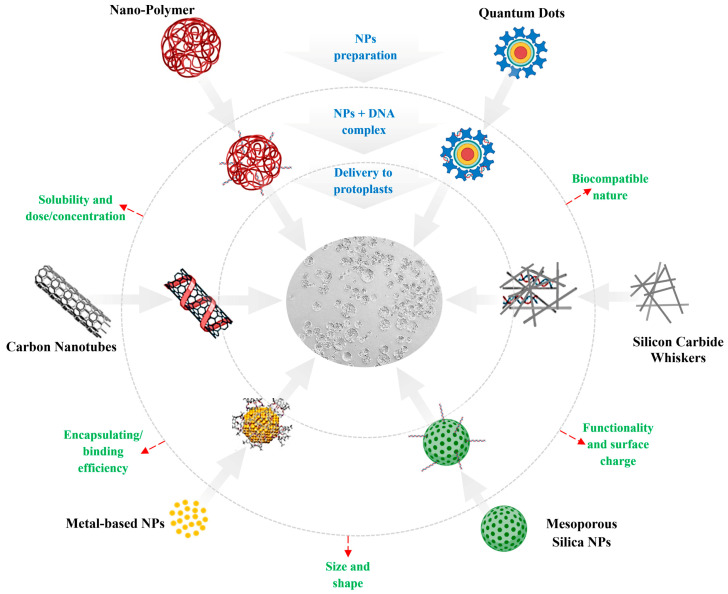
Graphical presentation of different nanomaterials as a suitable substitute for plant gene delivery systems. NPs are selected based on several key properties to optimize their efficacy in biomolecule delivery, such as biocompatibility, encapsulation/binding efficiency, solubility, size, shape, charge, and surface properties. The figure was created with BioRender.com.

Although NPs offer several potential benefits over traditional methods, achieving high transformation efficiency remains challenging. Transformation efficiency is affected by NP size, shape, surface properties, and delivery methods (Figure 1).

Smaller nanoparticles with a negative charge typically have greater uptake by cells. Rod-shaped NPs tend to enter some cells more efficiently than spherical NPs [22,23]. However, uptake efficiency depends on the target cell’s species and tissue source (Table 1). Thus, nanomaterials should be customized for each target cell or tissue, and transformation protocols should be optimized for maximum efficiency but minimum cytotoxicity.

**Figure 2:**
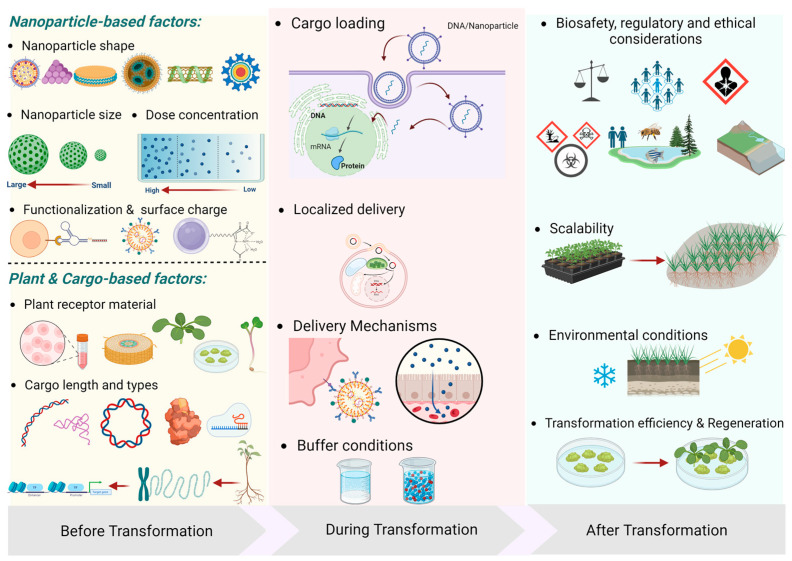
Overview of the key steps in developing a successful protocol for nanoparticle-based plant transformation. The process can be broken down into three major phases: before, during, and after transformation. Before transformation, the intrinsic properties of nanoparticles greatly influence their efficacy. The properties of the plant and the cargo must also be considered. During transformation, the exposure time, buffer conditions, and the amount of cargo loaded into the nanoparticles will be important for the process outcome. After transformation, the plant cells or tissue will need to be propagated, which can have biosafety and environmental consequences. The figure was created with BioRender.com.

Furthermore, as mentioned earlier, fabricating material at the nanoscale can alter chemical properties, which may significantly impact target cells. NPs may lead to cellular stress or toxicity, potentially impacting cell viability and transformation efficiency. Therefore, to develop a successful protocol for a plant-based system, three key areas need to be optimized—before transformation, during transformation, and after transformation (Figure 2).

In the sections below, these complex parameters will be examined as they influence plant transformation.

### 2.1. Before Transformation

#### 2.1.1. Plant and Cargo-Based Factors

Certain critical parameters of cargoes may require optimization when using NPs for DNA/RNA delivery to different plant tissues and successful genetic engineering and biotechnology applications.

*Plant material*. The limit of what can pass through the cell wall can differ depending on factors like plant species, cell type, and the unique composition of the cell wall [41,42]. However, it is typically estimated to be in the range of 20–50 nm [39]. The composition and structure of the plant cell wall exhibit significant variation not only between different plant species but also within different tissues of the same plant [43,44]. Some plant species have more porous cell walls that allow for easier NP penetration, while others may have more rigid and resistant walls [12]. The variability in cell wall permeability complicates the delivery of genetic material to an entire plant or multiple tissues simultaneously. This challenge would be particularly relevant when creating a stably transformed plant that expresses an altered gene in every tissue. For this reason, removing the cell wall entirely—such as in the case of protoplasts—can be advantageous. The hydrodynamic size limit for efficient NP delivery into leaf cells was determined to be 20 nm for cotton and 11 nm for maize, suggesting differences in the cell wall pore size between plant species [37]. The target cell type can significantly influence the effectiveness of NP delivery in plant systems. This variability arises from differences in cell membrane characteristics, receptor expression, and physiological functions. Different tissues from the same plant can be used as targets of transformation, usually in the form of explants. The selection of explants depends upon factors such as regenerative capacity, ease of handling, and the specific objectives of the transformation experiment [45]. In some plants, regeneration-competent cells originate from pre-existing undifferentiated meristematic cells because these cells have a simple and efficient regeneration process. Cotyledonary nodal regions in soybeans, known for their axillary meristems, are commonly used as the main explant because they offer high regeneration capacity and are easy to manipulate [46]. For example, the genetic transformation of hypocotyls from soybeans and canola is efficient, relatively easy, and fast, which has made them useful in research and agricultural applications [47,48].

In many other plant species, regeneration-competent cells originate from reprogrammed differentiated somatic cells through a dedifferentiation process that allows somatic cells to regain the capacity for proliferation competence or pluripotency [49]. For example, immature embryos are the main method for transformation in plants such as wheat and rice [50]. Immature embryos possess cells that can undergo dedifferentiation and subsequent regeneration, making them suitable targets for genetic transformation.

*Cargo lengths and types*. Delivering different cargos, including plasmid DNA and DNA-free gene editing reagents, such as ribonucleoproteins (RNPs) or mRNA and proteins, is possible using NPs. For example, SWCNTs are effective at delivering plasmid DNA and facilitating transient gene expression in somatic cells. In contrast, rosette nanotubes (RNTs) are often preferred for microspore delivery due to their ability to penetrate the cell walls of viable microspores. Additionally, BioClay is particularly advantageous for RNA delivery applications because of its robust protective qualities and durability [51,52,53].

NPs have been used to successfully deliver DNA lengths ranging from 20 to 15,000 bp to plant cells [36,54]. Moreover, the upper limit for plasmid size delivered by NPs in plant cells may continue to evolve as new research and advancements emerge. Due to their ability to complex with nucleic acids, single-wall carbon nanotubes (SWCNTs) have been used to deliver small interfering RNA (siRNA) and, more importantly, plasmids up to approximately 10 kb [31,55]. In the case of proteins, while studies have documented the absorption of certain large 100 nanometer-engineered NMs into plant cells with walls [56,57], there are indications of a selective barrier for proteins with a size below 10 nm or a molecular weight of approximately 100 kDa [58].

#### 2.1.2. NP-Based Factors

*NP shape and size*. The size and shape of NPs can affect their ability to enter plant cells. Since nanomaterials intrinsically have a high surface area relative to volume, they provide several advantages. A larger surface area than the same number of bulk materials of NPs can provide several advantages and unique properties for gene delivery into plants, including efficient cargo loading, enhanced cellular uptake, surface functionalization, and controlled release [59,60].

The plant cell wall’s rigid structure acts as a physical barrier that restricts the penetration of biomolecules [61,62]. The size of nanocomplexes (100 to 200 nm) used for nucleic acid transformation poses a challenge when facing the plant cell wall’s size exclusion limit (3 and 10 nm in diameter) [63,64]. Since they can better penetrate cell walls, smaller nanostructures are more efficient at delivering nucleic acids into plant cells. CNTs can potentially penetrate cell walls due to their small size and unique physical properties, making them interesting candidates for such applications [31,33,38,65]. Delaminated layered double hydroxide lactate (LDH-lactate-NS), which are positively charged nanosheets, vary in thickness from 0.5 to 2 nm and in diameter from 30 to 60 nm. Since they are so thin, they are highly effective at transporting target cargo across the cell wall barrier in intact plant cells [39].

The maximum size of NPs that can freely penetrate plant pollen cells is approximately 50 nm; larger NPs, approximately 30–50 nm in size, can overcome size exclusion limits and be internalized or translocated into plant cells by interacting with the plant cell wall in ways that lead to structural changes, pore enlargement, and disruption of actin filaments [66,67]. In contrast, the cell membrane, the lipid bilayer surrounding the cell, has a larger size exclusion limit of around 300 to 500 nm. This larger size exclusion limit allows for the passage of larger cargo into the plant cell through the cell membrane and smaller NPs through the cell wall [43,68,69]. NPs with a cylindrical shape (such as CNTs) and high tensile strength (for maintaining the structural integrity of carriers) can bypass the plant cell wall [55,70].

*DNA/NP ratio*. The DNA/NP ratio is crucial for optimal gene delivery. The ratio should be carefully adjusted to ensure sufficient genetic material is delivered into the cells without overwhelming or saturating the NP carriers. Torney and colleagues used MSN as carriers for DNA delivery into tobacco mesophyll protoplasts [8]. MSNs are NPs characterized by their large surface areas (>800 m^2^ g^−1^) and tunable pore sizes (2–10 nm in diameter). The study demonstrated that a DNA/MSN coating ratio of 1/10 (*w*/*w*) was optimal for forming a stable DNA-MSN complex, as indicated by the lack of free DNA in the solution after 2 h of incubation.

*Dose/concentration of NPs*. At high concentrations, nanomaterials can cause oxidative stress by inducing the production of reactive oxygen species (ROS), which can damage cellular structures and disrupt normal cellular functions [14,71]. ROS accumulation can lead to a significant loss of cell viability. For instance, the exposure of plant cells to high concentrations of AgNPs, silver NPs, caused a significant increase in ROS and loss in cell viability, which resulted in detrimental effects on rice (*Oryza sativa* L.) seedlings [19].

The cytotoxicity of NPs depends on multiple factors, including their type, size, shape, surface properties, chemical composition and concentration, exposure time, and the plant species, as well as the nature of their interactions with plants [12,51]. Assessment of cytotoxicity in plant cells is either qualitative or quantitative. Qualitative cytotoxicity assessments include microscopic observation of cell morphology and reactivity zones. Reactivity zones refer to areas of the plant tissue that show visible changes in response to nanoparticle exposure, such as discoloration, cell death, or other signs of stress, indicating the cytotoxic effects of the nanoparticles. Finiuk et al. and Van Doorn et al. estimated the cytotoxic effect of oligoelectrolyte polymer carriers by counting damaged and normal protoplasts using morphological criteria in Allium cepa and *Nicotiana tabacum* protoplasts [72,73]. Quantitative cytotoxicity measurements involve analysis of the expression of genes associated with stress and viability [74]. Demirer et al. used quantitative PCR analysis of the stress-responsive gene respiratory burst oxidase homolog B to evaluate the cytotoxicity of a CNT-based approach in tobacco plants [31].

*Functionalization and surface charge*. Functionalization of NPs refers to the modification of the surface or interior of NPs with specific functional groups, molecules, or ligands. These functional groups can target the NPs to specific cellular compartments, such as the nucleus and chloroplasts [33,75]. These functional modifications can also include the incorporation of biodegradable amino-ester lipids into the NPs for CRISPR/Cas9 delivery. By modifying the functional groups on the ester chains, the rate of biodegradability can be controlled, with steric effects slowing down the degradation rate. This approach helps improve the stability and delivery efficiency of CRISPR/Cas9 cargoes [76]. Future research should optimize nanoparticle chemistries for loading CRISPR reagents, such as covalently attaching Cas9 ribonucleoproteins (RNPs) to nanoparticles with enzymatically cleavable linkers and improving delivery efficiency to enable practical genome editing in plants [54]. These functional modifications rely on an extensive understanding of plant cell biology to ensure that NPs are efficiently administered and internalized by the appropriate cells. The most challenging aspect of this approach is that functionalizing poly lactic-co-glycolic acid (PLGA) NPs is difficult because of their relatively hydrophobic nature, limited surface chemistry, and potential for instability during modification, but functionalizing gold NPs is easier because of their ease of preparation and conjugation, biocompatibility, good tunability, and high stability (Table 1) [77,78].

Some functionalization strategies have attached proteins or targeting molecules to the surface of NPs, which can enhance cellular uptake or guide target genetic material to specific target cellular locations [79]. In particular, cell-penetrating peptides are an interesting way for NP functionalization due to their evolutionary design, which enables them to effectively penetrate host cell walls and membranes. For example, a synthetic peptide with a D-arginine-rich domain (dTat: rrrqrrkkr) has been shown to enhance the cellular uptake and cytosolic translocation of a DNA–polycation-peptide complex in plant protoplasts [80]. Similarly, a dual-domain cell-penetrating peptide (CKXAKXAKXAGWWG-NH2, X = α − aminoisobutyric acid (Aib)) has been utilized to deliver DNA to cell nuclei. Furthermore, combining cell-penetrating peptide-functionalized micelle complexes with chloroplast-targeting peptides has been demonstrated to successfully direct DNA to chloroplasts [42].

The surface tension and surface charge of NPs are dependent on the binding affinity between the cargo (such as genes or other biomolecules) and the NP matrix. Thus, it should be noted that although the functionalization of NP surfaces has been the focus of NP design, cargo content also influences transformation efficiency [18]. Therefore, if functionalization changes the surface charge of an NP, it can change how the NP adheres to the plant cell surface or enters the cell. The surface of root cells has a negative charge that allows positively charged NPs to be readily absorbed by plant roots [15,81]. The zeta potential of NPs is also an important factor in the stability of NPs in aqueous mixtures, which can be important in field studies where NPs are often administered as water-based mixtures. For this reason, particles with zeta potential higher than +30 mV or lower than −30 mV are considered stable [82,83]. Lipid membranes vary in their electrostatic charges based on their lipid and protein compositions, which affect their tendency to bind and internalize into cells. For example, plant cell membranes internalize NPs with a zeta potential above 20 mV, but chloroplast membranes internalize NPs above 30 mV, and neutral NPs (between −10 and +10 mV) are unlikely to penetrate any lipid bilayers [10,84].

It is also important to consider the surface tension of hydrophilic NPs, especially those with a hydrodynamic size larger than 2 nm, because it has been demonstrated that the use of surfactants, such as Silwet L-77, which reduces the surface tension of NP formulations (~22 mN/m), increased their uptake (<10 min) through leaf stomata and cuticle pathways [37].

### 2.2. Transformation

#### 2.2.1. Buffer Conditions

The choice of buffer or medium is crucial in NP dispersion and delivery systems, particularly when dealing with biological systems like plant cell cultures. The colloidal stability of NPs is influenced by the ionic strength and the osmotic concentration of the buffer, and NP compatibility with the chosen buffer is essential for maintaining cargo stability and NP functionality [85]. In optimizing the buffer for NP dispersion and delivery in plant cell cultures, it is often necessary to conduct compatibility studies whereby the stability of the NPs under conditions that mimic the plant cell culture environment is assessed. Based on these biocompatibility studies, it may be necessary to modify the NP surface (e.g., charge, elasticity, and NP synthesis) or change the cargo composition (e.g., buffer conditions, the ratio of DNA to NP, exposure duration, and sterility).

#### 2.2.2. Delivery Mechanisms

**Figure 3:**
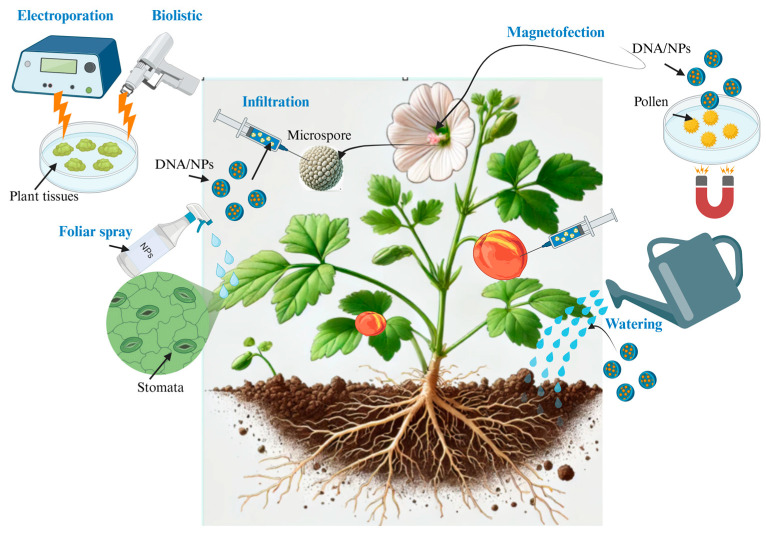
Summary of various delivery methods of nanoparticles and genetic material introduced in the plant cells. The figure was created with BioRender.com.

Identifying the most efficient and reliable delivery mechanisms to overcome various physiological and chemical barriers for NPs is crucial [15,16]. To better examine the challenges faced by plant biotechnologists, one can consider gene delivery to a plant leaf—or foliar gene delivery (Figure 3). Foliar spray involves applying substances directly to plant leaves, thereby delivering the cargo in the form of fine particles or droplets suspended in air and allowing for large-scale applications of transformation [86]. The efficient delivery of NPs into leaf tissue is dependent on the pore size of the stomata and leaf cuticles, cuticle composition, NP surface properties, and NP adhesion to leaves, a balance between the hydrophobicity and hydrophilicity of leaf surfaces.

Delivering genetic materials to the interior of intact plant leaves can be challenging because it is necessary to transform millions of plant cells simultaneously. Furthermore, this type of transfection does not necessarily lead to transformation in the traditional sense. Instead, it results in transient expression, where the gene constructs are temporarily active within the plant cells. Transient expression enables the temporary activation of gene constructs in plant cells without causing permanent changes to the plant’s genetic material. This temporary expression can be useful for various purposes, including accelerated studies of gene function, protein localization, and screening for gene activity within a relatively short timeframe, typically ranging from 2 to 10 days after delivery of the gene construct [87,88].

Indeed, most NP-mediated delivery techniques do not yield a stable transformation. However, several successful instances of stable transformation via NP delivery systems have been reported. These include the magnetofection of cotton pollen (Figure 3) [36], utilizing SWCNTs and multi-walled carbon nanotubes (MWCNTs) for protoplasts and cell-walled plant cells of tobacco [32], and employing mesoporous silica NPs (MSNs) for maize callus [26]. There is a need to enhance these methods through integration with novel technologies to produce stably transformed transgenic plants.

Another challenge in delivering genetic materials to the interior of the plant leaves is the presence of the waxy cuticle, a hydrophobic barrier that covers the leaf surface. Bypassing the epidermis and cuticle barriers to access the plant’s inner tissues is critical to many plant research and biotechnology applications. The cuticle’s pores typically have diameters less than 5 nm, making it difficult for most molecules, including NPs and genetic vectors, to pass through. Yet, many approaches have used passive delivery (endocytosis and membrane penetration) to introduce NPs into the cell. Mechanical disruption techniques have used a needleless syringe (direct injection of NPs into plant cells), ultrasound, a magnetic field, or a gene gun (a biolistic particle delivery system) to overcome the barrier posed by the cuticle (Figure 3) [89]. For instance, a study reported using mesoporous silica nanoparticles (MSNs) for the stable transformation of tomato plants by directly injecting them into tomato fruits. This approach was proposed as a suitable alternative to conventional genetic transformation methods, offering advantages such as biodegradability, biocompatibility, cost-effectiveness, and time efficiency (Table 1) [5]. Less disruptive approaches, arguably gentler to the target cell, include vacuum infiltration, syringe infiltration, foliar surfactant aerosol spray, or developing chemical compositions that can penetrate the cuticle [90]. Each method offers distinct benefits and drawbacks, with the selection process influenced by factors such as the type of nanoparticles, the intended application, and the specific plant species being targeted. Vacuum infiltration is suitable for introducing substances into numerous plants simultaneously, making it practical for research involving multiple plant samples. In contrast, syringe infiltration provides more precise control over the delivery site within the leaf [91].

Transporting nanoparticles through plant roots involves overcoming several distinct barriers. Su et al. compared the efficacy of nanoparticle delivery via leaves versus soil. Their findings, supported by other studies, indicate that positively charged carriers tend to be sequestrated in the root tissue, limiting their movement to other plant parts, such as shoots or leaves, where their effects may be needed [92,93]. However, the increased affinity between negatively charged NPs and plant tissues can help negatively charged NPs to transport effectively within the plant [92,93].

#### 2.2.3. Cargo Loading

NPs can enter plant cells through several mechanisms, including diffusion, endocytosis, or direct uptake via ion channels and plasmodesmata transport channels [13,94]. The NPs are designed to protect the biomolecules from degradation by extracellular nucleases and facilitate their delivery into plant cells [89]. Once inside plant cells, the NPs release the encapsulated biomolecule through controlled release mechanisms or by break-down of the NP complexes [12]. The efficient loading of biomolecules (e.g., DNA, RNA, proteins, or chemicals) onto or into NPs can be challenging and depends greatly on the chemical properties of the NP and the cargo and whether they are compatible with one another. For example, it is easier to encapsulate negatively charged nucleic acids into cationic lipid NPs, but it is very difficult to encapsulate hydrophilic drugs into hydrophilic PLGA matrices [95]. Protocols for encapsulating or attaching cargo into NPs aim to create NPs that have a high payload and remain stable, although that is not often achieved when working with incompatible materials. Different types of NPs (chitosan, polyethyleneimine, protamine, carbon quantum dot, polyamidoamine, and chitosan/SPc complex) were used by Wang and colleagues as RNA carriers to enhance RNA silencing in rice plants (Table 1) [27]. They found that among the nanoparticles tested, the chitosan/SPc complex and carbon quantum dot were the most effective for RNA encapsulation, with the chitosan/SPc complex enhancing dsRNA stability and uptake. The chitosan/SPc complex also prolonged the protective effect of dsRNA, reducing pathogen infection for up to 20 days, highlighting the importance of efficient cargo loading for improving RNA delivery.

#### 2.2.4. Localized Delivery

Off-target effects result in unintended genetic modifications and potentially harm the plant’s physiology. Localized delivery within rather inaccessible tissues remains challenging for plant and animal tissues. Investigating the movement of NPs within plants is essential for understanding their behavior and distribution. This research provides insights into their pathways, ultimate destinations, and the mechanisms involved in cargo release. Employing nanocarriers offers a versatile and effective means to protect the cargo from cellular metabolism and degradation and deliver biomolecules to specific cellular or tissue targets. This protection ensures that the cargo remains intact and functional until it reaches its intended destination, increasing the efficacy of various applications [96]. Carbon NPs carrying reporter genes have been used to transform dicot and monocot species for over 7–10 days [31,33]. Numerous studies have improved cargo delivery efficiency by adjusting the physical characteristics of nanoparticles and modifying the methods of cargo binding [37,51,86].

### 2.3. Post-Transformation

#### 2.3.1. Transformation Efficiency and Regeneration

One of the primary challenges of gene delivery, in plants or mammalian cells, is to achieve high transformation efficiency. Not all NPs effectively deliver genes into plant cells, and the rate of successful gene integration can be relatively low. Thus, improving the efficacy of this process is essential for practical applications [31].

NP-mediated delivery has the potential to overcome regeneration-specific challenges in plant genetic engineering by allowing for direct germline editing of plant tissues without the need for regeneration [36]. Bypassing regeneration is particularly crucial for plant species with long or difficult regeneration times, such as cotton. For example, by utilizing pollen magnetofection and directly introducing genetic material into pollen, Zhao and colleagues produced transgenic seeds without regeneration and bypassed the conventional process of plant regeneration from cotton tissue culture [36]. While NP-mediated delivery offers advantages, it still faces challenges related to the efficiency of delivery, the specificity of targeting, and potential off-target effects.

A plant’s genetic composition can influence delivery success because regeneration capacity is a genetically controlled trait that differs across plant species and even among cultivars within a species. This variation is driven by genetic factors and the regulatory networks that control cell differentiation, proliferation, and organogenesis, which affect a plant’s ability to be regenerated in vitro [45,47]. While some plant species like tobacco, Arabidopsis, and rice are known for their ease of regeneration in vitro, others, such as soybean, wheat, and maize, present greater challenges in tissue culture and regeneration [45]. Therefore, tobacco, Arabidopsis, and rice are generally considered good model systems for studying plant transformation, while researchers may face more difficulties when working with soybean, wheat, and maize, requiring additional efforts in developing efficient transformation protocols. Genotype dependence in plant transformation limits successful genetic transformation to specific plant species or varieties, with protocols often not transferable between varieties. To address these challenges, strategies have been developed to overcome recalcitrance, or low regeneration capacity, in tissue culture. These include: 1) using genotype-independent explants like meristematic tissues, 2) applying plant hormones to pretreat explants before gene delivery, and 3) overexpressing morphogenic transcription factors to enhance regeneration.

#### 2.3.2. Environmental Factors Affecting the Transformed Plant Growth Using NPs

Environmental factors are important in determining the success of plant transformation and their subsequent growth, especially when NPs are involved. Several key environmental factors influence transformed plant growth using nanoparticles, including plant growth regulators, light, temperature, and the type and concentration of nanoparticles. The presence and concentration of plant growth regulators can be altered by the addition of nanoparticles, which may interact with plant growth regulators and either enhance or inhibit their effects. Light and temperature are critical environmental factors influencing plant growth, especially in in vitro conditions like tissue culture. It can affect photosynthesis, plant morphogenesis, metabolic processes, enzyme activity, and the overall health of transformed plants.

Nanoparticles have unique physicochemical properties that can influence plant growth, gene expression, and overall health, but their effects are heavily modulated by various environmental conditions. The duration of exposure to NPs and the timing of delivery with the plant’s growth stage can affect uptake [27]. Potential negative consequences of long-term exposure of plants to polymer NPs include a reduction in germination rates due to the blockage of pores and subsequent impairment of nutrient absorption [97]. Optimization may involve determining the ideal exposure time for each tissue type. For instance, the germination rate of wheat seeds and onions was not affected when treated with polystyrene nanoplastics for 72 h [98,99], while significant decreases in plant seedling growth—by 50% in *Arabidopsis thaliana* after 30 days and by 41.5% in onion after seven weeks—have been reported as a result of long-term exposure to NPs [100,101]. Therefore, environmental factors are deeply interconnected with the presence and concentration of nanoparticles in plant systems. The combination of plant growth regulators, light, temperature, nanoparticle type, and concentration must be carefully balanced to optimize the transformation process and promote the healthy growth of genetically transformed plants.

#### 2.3.3. Scalability

**Figure 4:**
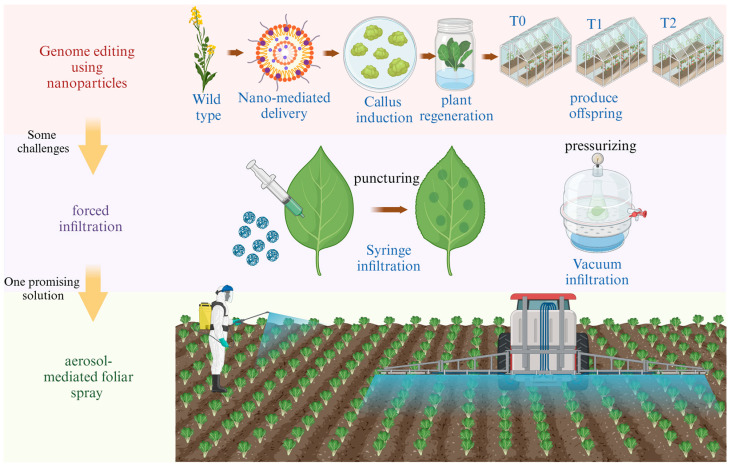
Scalability challenges and solutions in NP-mediated gene transformation: from tissue culture bottlenecks to aerosol-mediated foliar spray. The figure was created with BioRender.com.

Scaling up NP-mediated gene transformation for large-scale agricultural production can be challenging because of issues such as a significant bottleneck in scaling regeneration from tissue culture [102] and the obligatory use of puncturing and pressurizing for the infiltration of NPs into leaves (Figure 4) [31]. Additionally, the hormone and growth medium requirements for somatic cell differentiation in tissue culture are often undefined for many species, and the mutagenic process of cell passaging can take months to years. Most genetic engineering techniques usually focus on immature, undifferentiated tissues, such as callus or meristems, which necessitate expensive and time-consuming tissue culture procedures to produce offspring. Developing cost-effective methods that can be applied to various crops is important for practical implementation. One promising solution is using aerosol-mediated foliar spray with nanoparticles (Figure 4), which provides an efficient method for large-scale cargo delivery in plant transformation. This approach bypasses the need for tissue culture and offers a more direct and potentially faster method for gene editing, which could significantly reduce both time and cost in crop improvement programs.

#### 2.3.4. Biosafety, Regulatory, and Ethical Considerations

The application of NPs in plant biotechnology presents potential safety risks that must be carefully considered. NPs can enter biological systems through the ingestion of food, soil, and water, leading to acute and chronic exposure risks for humans, plants, and other organisms [21]. Studying the fate of NPs and/or functionalized materials after cargo molecule delivery is crucial to assessing their biocompatibility, biosafety, and potential toxicity [14]. As with any genetic modification technology, there are regulatory and ethical considerations surrounding NP-mediated plant transformation. Ensuring that technology complies with regulations and addresses public concerns is essential. Transient expression of target biomolecules presents a promising avenue for creating transgene-free genome-edited crops via NP-mediated delivery [86]. The absence of foreign DNA in the final edited crops may circumvent the need for labeling as genetically modified organisms (GMOs) in many countries [27]. This absence of foreign DNA could address concerns related to consumer acceptance and regulatory requirements, making the commercialization of edited crops more feasible. The use of peptide and chemical functionalization of NPs is a versatile strategy that has been explored in mammalian cells [103,104] and may have potential applications in plants. This approach could mitigate concerns about toxicity and contamination associated with using NPs in plant applications.

Several strategies can be adopted to ensure the safe and responsible use of nanomaterials in plant biotechnology. First, comprehensive risk assessments should be conducted to evaluate the potential hazards associated with NP use. These assessments should include toxicity studies, environmental impact analyses, and long-term monitoring. Second, best practices for NP use should be developed, including guidelines for safe handling, application rates, and disposal methods. Third, interdisciplinary collaboration among scientists from different scientific areas, regulators, and industry stakeholders is essential to address the complex challenges of NP applications. By adopting these strategies, we can harness the benefits of NPs in plant biotechnology while minimizing potential risks to human health and the environment.

## 3. Future Perspective

NP-mediated gene delivery has the potential to revolutionize agricultural biotechnology, but significant challenges remain in developing efficient, low-toxicity, and species-independent transformation protocols. A major area for future advancement is the design of next-generation nanocarriers. Tailoring nanomaterials with specific properties, such as size, shape, surface charge, and functionalization, will be essential for optimizing gene delivery across various plant species and tissues. Although substantial progress has been made, much remains to be carried out in understanding the intricate interactions between NPs and plant cells.

Looking ahead, innovations in nanomaterials with reduced side effects will significantly enhance plant biotechnology. These advancements can address challenges in grain production and predictive breeding, facilitating the development of crops with improved traits. Integrating nanotechnology-enhanced genetic engineering with genomic selection, as demonstrated in the successful genomic prediction of wheat landraces, could accelerate the application of nanotechnology in improving cultivated gene pools and advancing agricultural productivity [105]. Moreover, the future success of nanoparticle-mediated transformation will depend on addressing the delicate balance between effective cargo delivery and minimizing adverse effects like oxidative stress. Additionally, with the integration of cutting-edge technologies, such as CRISPR/Cas9, there is the potential for even greater precision in plant genetic manipulation, opening up new avenues for crop improvement and the production of valuable biomolecules. Despite successful DNA and protein delivery into plant cells using nanomaterials, CRISPR-Cas genome editing has not yet been achieved due to challenges in loading large CRISPR plasmids and overcoming delivery inefficiencies with Cas9 protein.

Future experimental approaches should focus on addressing critical gaps in NP-mediated gene delivery. For instance, testing the efficacy of nanoparticles across diverse plant species will be essential for developing species-independent transformation protocols. Additionally, exploring biodegradable nanomaterials can improve biosafety and reduce potential environmental impacts, ensuring that these technologies are both effective and sustainable. Another promising avenue is the design of nanocarriers capable of multiplex gene delivery, which could enable the simultaneous delivery of multiple genetic cargos, enhancing the efficiency and versatility of plant genetic engineering. These experimental directions will help address existing challenges and accelerate the development of robust NP-based gene delivery systems.

Interdisciplinary approaches that combine nanotechnology, plant biology, and computational modeling hold tremendous promise for addressing these challenges. For example, computational models can simulate NP–plant interactions to predict optimal NP designs, while plant biology insights can guide the development of species-specific transformation strategies. Collaborative research across these disciplines can lead to breakthroughs in designing more efficient and targeted gene delivery systems, as well as improving our understanding of the underlying biological mechanisms.

Lastly, ethical considerations and environmental impacts will need to be carefully evaluated as NP technologies are adopted on a wider scale. Understanding the potential environmental risks and long-term effects of nanoparticles in ecosystems will be crucial to ensuring that their use in plant biotechnology remains both safe and sustainable. Regulations via legislation, laws, and rules have been implemented by several regulatory agencies and government organizations, such as the Food and Drug Administration, the Environmental Protection Agency, and the European Food Safety Authority, to control the potential risks of NPs [20,106]. However, these guidelines often lack specificity for agricultural applications and fail to address the unique challenges NPs pose. NPs exhibit variations in shape, size, chemical composition, and physicochemical properties, making it crucial to understand which specific profiles can cause negative health impacts [107]. Updated policies are urgently needed to integrate the latest scientific discoveries and offer clear guidance on the safe and responsible application of nanomaterials in plant biotechnology. In general, NPs have minimal toxicity, which may yield more opportunities for future agricultural development [108]. Many NPs are biocompatible with plant tissues and protoplasts [109,110].

## 4. Concluding Remarks

In conclusion, nanoparticle-based gene delivery systems are transforming plant biotechnology by offering an innovative, efficient, and flexible approach to genetic transformation. These systems present a wide range of possibilities for genetic engineering, crop improvement, and the development of sustainable agricultural practices. However, several challenges remain, including the complexity of plant-specific barriers to NP delivery, the optimization of NP properties for different plant tissues, and the minimization of potential cytotoxicity.

In addition to optimizing NP carriers, understanding the biological factors influencing NP-mediated gene delivery is essential. These include tissue-specific characteristics that affect the efficiency of transformation. Research into plant regenerative capacities, such as callus formation, offers a potential solution for overcoming the transformation limitations of certain species. Callus tissue can facilitate NP transfer, bridging the gap for genetic transformation in species with poor regeneration capacities. Furthermore, a better understanding of the genetic factors that enable plants to internalize and respond to foreign genetic material will be essential for developing species-specific and universal transformation protocols. Materials like single-walled carbon nanotubes (SWCNTs), rosette nanotubes (RNTs), and BioClay have shown promise for RNA and plasmid DNA delivery, offering unique solutions for challenging plant tissues, particularly in species with low transformation efficiency [85].

Standardizing nanoparticle preparation, gene loading, and delivery protocols is required for enhancing reproducibility across studies and accelerating progress in nanoparticle-mediated gene delivery to plants. Consistent synthesis methods, such as optimizing reaction conditions for nanoparticle uniformity, have been established for silica and gold nanoparticles, ensuring reproducible properties like size and surface charge [111]. Similarly, well-defined gene-loading protocols, such as electrostatic binding with chitosan-coated nanoparticles, have demonstrated reliable nucleic acid loading for effective delivery [33]. Delivery methods, including biolistic gene transfer, benefit from standardized parameters, such as nanoparticle-to-DNA ratios, helium pressure, and target distances, to improve efficiency [111]. Agroinfiltration techniques combined with nanoparticles also rely on defined conditions, such as infiltration pressure and incubation time, to ensure consistent results [54]. By adopting and refining these protocols based on insights from recent studies, researchers can build a robust framework to streamline nanoparticle-mediated gene delivery and address current challenges in plant biotechnology.

The adoption of nanoparticle-based gene delivery methods also brings concerns regarding biosafety and plant toxicity. NP-mediated genome editing may result in off-target effects, requiring thorough evaluations of potential risks to human, animal, and environmental health. Regulatory frameworks, such as the European Directive on GMOs, necessitate long-term monitoring of GMOs to assess their environmental and health impacts. Similar monitoring efforts will be critical for nanotechnology applications in agriculture. Given the vast quantities of natural or artificial nanoparticles in the environment, it is important to carefully examine their potential detrimental effects, which have been reviewed extensively in the literature [95].

Overall, looking to the future, NP transformation holds immense promise for the agricultural sector. As technology advances, it is likely to lead to more efficient and sustainable farming practices, enhance the genetic potential of crops, and allow for more precise control over growth and production. The future of NP transformation in agriculture is improving crop yields and creating more resilient, nutritious, and environmentally sustainable food systems. By embracing this technology, we could see a future where agriculture is more efficient, resilient to climate change, and capable of feeding a growing global population. Whether it is through improved crop protection, enhanced nutrient delivery, or the development of high-value crops, NP transformation could become a cornerstone of next-generation agricultural innovation. However, achieving its full potential will require continued research, collaboration, and careful consideration of its ethical and environmental implications.

## Figures and Tables

**Table 1:** A comparative summary of the key properties, applications, and limitations of nanoparticles used in plant gene delivery.

Nanoparticle Type	Charge	Morphology	Functionalization	Applications in Plants	Advantages	Limitations	Delivery Methods	References
Gold Nanoparticles (AuNPs)	Positive/Neutral	Low aspect ratio (spherical)	PEGylation, thiol-functionalized	Gene delivery to *Nicotiana tabacum* and *Oryza sativa* (rice)	High tensile strength, ease of preparation and conjugation, biocompatibility, good tunability, and high stability	High cost, potential phytotoxicity, and instability during modification	Injection	[24,25]
Silica Nanoparticles (e.g., MSN)	Neutral/Negative	Spherical, porous structure	Amine-functionalized	Delivery of CRISPR/Cas9 and DNA to rice, maize, and tomato (*Solanum lycopersicum*)	High biocompatibility, tunable porosity, and high tensile strength	Complex synthesis and potential for environmental persistence	Spraying, injection, and gene guns	[5,8,26]
Chitosan Nanoparticles	Positive	Spherical	PEGylation	DNA delivery to *Triticum aestivum* (wheat) and RNA silencing in rice	Biodegradability and biocompatibility, low toxicity, enhancing dsRNA stability and uptake	Limited efficiency in some plant species	PEG transfection and co-culture	[27,28]
Polymeric Nanoparticles (e.g., PLGA)	Negative	Spherical- or needle shape	PEGylation, ligand functionalization	Delivery of siRNA to tobacco protoplasts and CRISPR/Cas9 mutagenesis in maize protoplasts	Biodegradable, scalable synthesis	Complex synthesis, low transfection efficiency, and self-aggregation	Injection or co-culture	[11,29]
Lipid Nanoparticles	Neutral/Positive	Core–shell structure	Lipid functionalization, PEGylation	Delivery of CRISPR/Cas9 in maize and citrus plants	Chemical diversity and functional potential, flexible structural designs, effective endosomal escape	Stability issues, limited loading capacity		[30]
Carbon-Based Nanoparticles (e.g., SWCN)	Neutral/Negative	High aspect ratio, cylindrical	Functionalizes with chitosan or PEI	DNA, siRNA, chloroplast-selective gene delivery in tobacco, spinach, arugula, and watercress	High cargo capacity, good cellular uptake, and high tensile strength	Potential toxicity, challenges with biodegradability; difficulty of imaging	Injection or co-culture	[31,32,33,34]
Magnetic Nanoparticles	Positive/Neutral	Spherical, cubic, rod	Amine and thiol-functionalized	Targeted gene delivery to rice and directly introducing genetic material into *Brassica napus* (canola) and cotton pollen	Targeted delivery under magnetic fields, ease of separation	Limited biocompatibility, potential aggregation	Magnetic field	[35,36]
Carbon dots (CDs)	Positive/Neutral	Low aspect ratio (spherical)	Functionalizes with PEI and PEG	Chloroplast delivery in cotton and maize; delivery of siRNA in *Nicotiana benthamiana* and tomato	Ease of synthesis and functionalization, minimal toxicity, and high biocompatibility	Difficulty of imaging	Low-pressure spray and foliar delivery	[37,38]
layered double hydroxide (LDH)	Positive	Hexagonal platelet suspension cell	-	Delivery of DNA and dsRNA uptake in intact cells of *Arabidopsis thaliana* and *Nicotianatobacum*	Biodegradability and biocompatibility, low toxicity; excellent transporters to living cells; high tensile strength	Limited understanding of LDH nanoparticle internalization and intraplant distribution mechanisms	Passive delivery; topical spray	[39,40]

## Data Availability

No new data were created or analyzed in this study. Data sharing is not applicable to this article.

## Abbreviations

NMs: nanomaterials; NPs: nanoparticles; MSNs: Mesoporous Silica nanoparticle; RNTs: rosette nanotubes; CNTs: carbon nanotubes; SWCNTs: Single-wall carbon nanotubes; MWCNTs: multi-walled carbon nanotubes; carbon dots (CDs); layered double hydroxide (LDH).
